# How MALDI-TOF Mass Spectrometry Technology Contributes to Microbial Infection Control in Healthcare Settings

**DOI:** 10.3390/vaccines10111881

**Published:** 2022-11-08

**Authors:** Ayman Elbehiry, Musaad Aldubaib, Adil Abalkhail, Eman Marzouk, Ahmad ALbeloushi, Ihab Moussa, Mai Ibrahem, Hamad Albazie, Abdullah Alqarni, Sulaiman Anagreyyah, Saleh Alghamdi, Mohammed Rawway

**Affiliations:** 1 Department of Public Health, College of Public Health and Health Informatics, Qassim University, Al Bukayriyah 52741, Saudi Arabia; 2 Department of Bacteriology, Mycology and Immunology, Faculty of Veterinary Medicine, University of Sadat City, Sadat City 32511, Egypt; 3 Department of Veterinary Medicine, College of Agriculture and Veterinary Medicine, Qassim University, Buraydah 52571, Saudi Arabia; 4 Al Bukayriyah General Hospital, Qassim, Al Bukayriyah 52725, Saudi Arabia; 5 Department of Botany and Microbiology, College of Science, King Saud University, Riyadh 11451, Saudi Arabia; 6 Department of Public Health, College of Applied Medical Science, King Khalid University, Abha 61421, Saudi Arabia; 7 Department of Aquatic Animal Medicine and Management, Faculty of Veterinary Medicine, Cairo University, Cairo 12211, Egypt; 8 Department of Support Service, King Fahad Armed Hospital, Jeddah 23311, Saudi Arabia; 9 Department of Preventive Medicine, King Fahad Armed Hospital, Jeddah 23311, Saudi Arabia; 10 Department of Biomedical Engineering, King Fahad Armed Hospital, Jeddah 23311, Saudi Arabia; 11 Biology Department, College of Science, Jouf University, Sakaka 42421, Saudi Arabia; 12 Botany and Microbiology Department, Faculty of Science, AL-Azhar University, Assiut 71524, Egypt

**Keywords:** MALDI-TOF MS, healthcare settings, microbial identification, antimicrobial resistance

## Abstract

Healthcare settings have been utilizing matrix-assisted laser desorption ionization time-of-flight mass spectrometry (MALDI-TOF MS) since 2010. MALDI-TOF MS has various benefits over the conventional method of biochemical identification, including ease of use, speed, accuracy, and low cost. This approach can solve many of the obstacles to identifying bacteria, fungi and viruses. As technology advanced, more and more databases kept track of spectra, allowing species with similar morphological, genotypic, and biochemical traits to be identified. Using MALDI-TOF MS for identification has become more accurate and quicker due to advances in sample preparation and database enrichment. Rapid sample detection and colony identification using MALDI-TOF MS have produced promising results. A key application of MALDI-TOF MS is quickly identifying highly virulent and drug-resistant diseases. Here, we present a review of the scientific literature assessing the effectiveness of MALDI-TOF MS for locating clinically relevant pathogenic bacteria, fungi, and viruses. MALDI-TOF MS is a useful strategy for locating clinical pathogens, however, it also has some drawbacks. A small number of spectra in the database and inherent similarities among organisms can make it difficult to distinguish between different species, which can result in misidentifications. The majority of the time additional testing may correct these problems, which happen very seldom. In conclusion, infectious illness diagnosis and clinical care are being revolutionized by the use of MALDI-TOF MS in the clinical microbiology laboratory.

## 1. Introduction

Through using conventional techniques, identifying bacteria and fungi can be a difficult and time-consuming operation [1,2]. As part of the analysis of bacteria and yeasts, phenotypic and molecular techniques can be used in addition to colony and Gram-stain morphology [3]. As regards fungi, species are frequently identified by their distinctive visible and microscopic examination. Bright-field microscopy was initially developed for the examination of adherent oral microorganisms [4]. This approach, however, produced reliable findings only with a suitable sample thickness. In an interesting development, the methodology for increasing the sensitivity of microscopy has been altered as a result of research on the biofilms produced by diverse bacteria [5]. The preparation of samples can be complicated, and result in artifacts in electron microscopy (EM), according to Hannig et al. [6], D’Ercole et al. [7], and Bernardi et al. [8]. A fundamental change has occurred in the way microbiological samples are analyzed since confocal laser scanning microscopy was developed in the 1990s [5]. Furthermore, the high resolution of microscopy, specifically EM, also provides a significant contribution to microbial characterization, allowing the visualization of specific ultrastructural details of a microorganism [9].

In molecular or conventional methods for detecting microorganisms, it is often challenging to distinguish between species that are morphologically, biochemically, or genetically similar [3]. Because species identification typically requires the emergence of one or more sub-cultures, using these conventional procedures can increase the time it takes to diagnose a patient. Furthermore, the perception of phenotypic traits is frequently arbitrary, necessitating extensive skill and expertise for precise identification. Genotyping may be used if these conventional approaches fail to recognize a microorganism, although this frequently involves lengthy delivery times and contributes significantly to the cost [3,10,11]. Phenotypic as well as genotypic approaches have their advantages and disadvantages, but genotypic approaches are more reliable, reproducible, and discriminatory for a wide range of microorganisms than phenotypic approaches [12]. EM continues to play an important role in virology and bacteriology, although molecular methods have a significant impact. The EM can provide distinct, yet equally valuable information about infectious organisms. By using EM, antigens can be found on and in bacterial cells, and some microorganisms can be determined from biopsy samples [13]. However, EM has the major drawback of having low sensitivity for microbial research, particularly when it comes to nonculturable patient samples [14]. Therefore, EM will be effective in detecting pathogens accurately if it complements other investigation methods rather than competes with them. By using this combination approach, we will be able to get a better understanding of many new characteristics of both well-known and newly discovered organisms, as well as the diseases they cause [13].

Currently, matrix-assisted laser desorption-ionization time-of-flight mass spectrometry (MALDI-TOF MS) technologies are widely available [15,16,17] that are quicker, more affordable, often with greater precision than was formerly feasible. With MALDI-TOF MS, it is possible to identify microbes quickly and it has become an essential tool in clinical microbiology laboratories [18,19]. In accordance with the protein sequence revealed by each microbial species, a UV laser is used to desorb and ionize the bacterial and fungal biomass encased in matrix, frequently α-Cyano-4-hydroxycinnamic acid (C_10_H_7_NO_3_) [20,21]. With MALDI-TOF MS, particles are ionized, separated according to their mass-to-charge (*m*/*z*) ratio, and measured by their time of arrival at detectors [22,23]. Based on mass-to-charge values along the *x*-axis and intensity along the *y*-axis, spectra generated are compared with spectra from known microorganisms [3]. When compared to older approaches, this technology can reliably identify mycobacteria, yeast, and molds, along with various types of bacteria, typically at the genus and species [3,24].

This technology is also more accurate and rapid at identifying microorganisms (even those closely related) than conventional and molecular techniques. This objective was accomplished mostly as a result of the enhancement of sample processing techniques used before MALDI-TOF detection [25,26]. Mycobacteria and filamentous fungus are now among the pathogens that can be regularly detected by MALDI-TOF because of enhanced sample processing [25,27]. Species that are essentially similar to one another and those that are not in the database are exceptions to this rule [28,29]. Testing can frequently be carried out from the pure cultures (single fresh colony) that are available because of the low biomass requirement [3]. There are now extensive, up-to-date datasets with both widespread and unusual species having clinical relevance. This is an additionally important element in obtaining correct authentication. For physicians to start tailored antimicrobial therapy when required, or to stop using unneeded antimicrobials, rapidity is also of the utmost significance [19]. Depending on whether it is necessary to extract proteins from colonies, which has become less common as a result of the improving efficiency of the methodology, the detection of clinical pathogens from colonies can take anywhere from 5 to 45 min [19,30].

Furthermore, it is urgently necessary to develop quick and accurate techniques for antimicrobial resistance testing due to the prevalence of multi-drug-resistant bacterial strains, particularly in healthcare setting [31]. Consequently, several studies looked into the feasibility of using MALDI-TOF MS methodology to quickly identify antibiotic-resistant bacteria that were isolated from different infections along with the ability to identify harmful fungi that were resistant to antibiotics [31,32]. In this regard, MALDI TOF has been shown to be beneficial for the treatment of infectious diseases, to assist in epidemiologic investigations, and to contribute to the development of strategies to prevent and control infection [33]. This review will focus on clinical microbiology in spite of mass spectrometry’s wide range of clinical applications and discuss how this technique can detect antimicrobial resistance and quickly identify microbes.

## 2. MALDI’s Historical Evolution

Despite being invented in the 19th century, mass spectrometry (MS) was only used in the chemical sciences. Nevertheless, the introduction of MALDI in the 1980s improved the usability of MS to biological macromolecules as proteins [34]. As part of Hillenkamp’s contribution to the advancement of MALDI, he and his collaborators used MS to illustrate MALDI in 1985 [35]. John Fenn and Koichi Tanaka shared the 2002 Nobel Prize in Chemistry for developing soft desorption ionization techniques for studying biological macromolecules, including electrospray and soft laser desorption. In 1994, Cain et al. reported a method utilizing MALDI-TOF MS that used proteome analysis with C_10_H_7_NO_3_ as a matrix and was beneficial for quickly identifying microorganisms [36]. Although, pretreatment bacteria were still used at that time for MALDI-TOF MS. Eventually, it was revealed [37,38] that the approach may possibly be utilized immediately to fresh colonies established on MALDI target plates without particular preparation, opening up the door for practical applications of the technology [39]. Clinical biological labs did not record the results of using this technique until 2009, and the results of the experimental stage did not become clinically applicable until many years later [39,40]. It takes a long time for research lab data to be converted into clinical settings, as evidenced by the fact that the primary information from a laboratory was only available in 2009. As described by Tsuchida et al. [39], there are a number of causes for this evident postponement, which include: (1) worry that the protein varies adaptively in vivo and is highly swayed by cultivation conditions; (2) uncertainty regarding whether the discrepancies in MS spectra are completely compatible with currently known categorizations; (3) complete absence of a massive database; and (4) the reality that, from the bacteriologist’s viewpoint, microbiological characterization is a challenging task that necessitates a certain level of preparation in microbiology (as opposed to applications). For medical applications, several studies are also needed at time of verification. Since 2009, there have been more medical published papers each year, and clinical microbiology laboratories are becoming more interested in studying the MALDI-TOF MS.

## 3. The Workflow of MALDI TOF Mass Spectrometry

**Figure 1:**
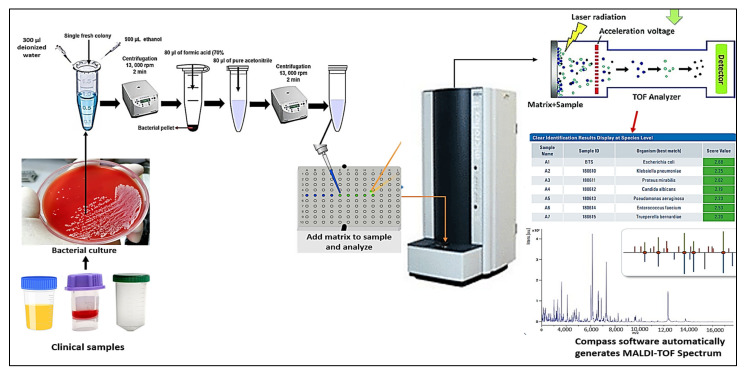
The workflow of MBT for identifying various microorganisms. The fresh colony is placed on a MALDI target plate that will be beamed in order for it to be identified by MS. The target plate is put into the device once the MALDI matrix has been added and processed with the unknown sample. The mass/charge ratio (*m*/*z*) of the constituent proteins is determined by the time of flight to the detector and the intensity of the signal. To ionize the ribosomal proteins molecules, the device irradiates the target plate with a laser. To identify the sample, the acquired MALDI mass spectral fingerprint is compared with the stored database.

As an analysis approach, MALDI-TOF mass spectrometry is dependent on the cellular protein, which represents the microorganisms’ genetic instructions and degradation products. This approach, in particular, is based on the investigation of highly expressed proteins, primarily ribosome, found in bacteria with masses between 2000 and 20,000 Daltons [41,42]. To determine the mass to charge (*m*/*z*) ratio, these proteins are ionized into charged molecules through the removal or addition of one or more protons. The sample is combined with an energy-absorbing mixture known as “matrix” for investigation. The sample trapped inside the matrix crystallizes along with it when it dries and solidifies. The sample is subsequently ionized by a laser beam, producing single protonated ions. Then, these ions are propelled at a set voltage and they split from one another according to their *m*/*z* ratio, which is calculated by timing how long it takes each ion to travel the entire length of the flight tube. The “Peptide Mass Fingerprint” (PMF), a distinctive mass spectral protein, is created using the time-of-flight data. Then, this PMF is matched to the library of the device. It contains signals unique to genera and species specific to particular types of microorganisms. The unknown pathogen is well-known at the genus, and species levels by comparing the PMF of unidentified bacterial isolates to those of stored in the database [34,42,43,44]. Today, MALDI-TOF MS has established itself as a reliable measuring method in microbial identification [42,43,45]. As previously indicated, the current microbial identification systems include the MALDI Biotyper (MBT, Bruker Daltonics, Bremen, Germany), the AXIMA (Shimadzu), and the VITEK MS (BioMerieux). Here, the fundamentals of analysis and use are explained through using MBT as an illustration (Figure 1). For the purpose of identifying bacteria and fungi, various specimen types have already been utilized or will be utilized in the future. Bacterial colonies from recent culturing are the most common sort of material. Periodically, new procedures are released in an effort to improve MALDI-identification TOF’s capabilities.

## 4. Implications of MALDI-TOF MS for Microbial Recognition

### 4.1. Bacterial Identification

The most common and best-optimized utilization of MALDI-TOF to date is for the regular recognition of clinical pathogens. Because of its incredible rapidity for microbial detection, whether from cultivation or directly from medical specimens, MALDI-TOF has during the past two decades become the standard method. The majority of microbial pathogens frequently seen in clinical microbiology facilities may now be successfully identified, employing MALDI-TOF due to an increase in the technique’s reliability over time [19,46]. The ability of MALDI-TOF to correctly identify species that are members of the Enterobacteriaceae (e.g., *Salmonella* spp. and *Cronobacter* spp.) at the genus level, according to a collaborative study completed in 2018 [47]. *Aeromonas*, *Citrobacter*, *Escherichia*, *Plesiomonas*, *Providencia*, *Serratia*, and *Yersinia*, also closed-related genera, were investigated in the same study, allowing the scientists to exclude *Salmonella* or *Cronobacter* confusion. It has been widely shown that MALDI-TOF can properly detect the most prevalent species of the Enterobacteriaceae [40,48]. Even so, it has presumably been revealed that MALDI-TOF misidentifies *Shigella* species as *Escherichia coli* and only partially distinguishes between those that belong to the *Citrobacter freundii*, *Enterobacter cloacae*, and *Salmonella enterica* species [40,49].

The practice of the majority of the microbiology labs currently includes the detection of non-fermenting Gram-negative rods by MALDI Biotyper. Various MALDI-TOF methods have been proven to properly identify the most frequent species seen in clinical settings (e.g., *Pseudomonas aeruginosa*, *Stenotrophomonas maltophilia*, and *Moraxella catharralis*) in 93.6–96.6% of the cases studied [19,50]. However, as a result of the similarity among the species in this genus, certain species have only been recognized on a broad scale (e.g., *Burkholderia cepacia* and *Acinetobacter baumannii*) or at a genus level (e.g., *Achromobacter* species, *Chryseobacter* species, and *Ralstonia* species). These species pose a problem for DNA-based techniques as well, but recognition at the species level is typically not important from a medical viewpoint. Updates in the library are recommended, utilizing reference strains accurately described by genetic approaches where recognition at the species level is necessary to determine the antimicrobial activities. Nevertheless, subsequent datasets and spectral analyzing advancements may make achieving a higher degree of reliability simpler [19].

When supplemented datasets are used, MALDI-TOF has also proved successful in identifying various Gram-negative bacteria, such as the HACEK group (*Haemophilus*, *Aggregatibacter*, *Cardiobacterium*, *Eikenella*, *Kingella*), with rates of 100% species precision [51]. In one investigation, a commercialized dataset produced 66.0% appropriate species level recognition but 93.0% proper genus level. This also applies to the *Neisseria* genus, because several species that are included in the normal microbial community have been mistaken for *Neisseria meningitides* [52]. The only way to avoid these mistakes and guarantee a correct assessment is through the usage of current and extended databases. As demonstrated in another category of this review, the level of reliability presently attained for the recognition of Gram-negative organisms via MALDI-TOF is still astonishing given that it is carried out in less than 1 h [53]. Oviaño and his colleagues [53] conducted a comparison study assessing the detection of suspected colonies from fecal samples by MBT compass software vs. conventional morphological approaches. They discovered that the full MALDI identification process, from preparation of the sample to obtaining the final result, was finished in less than 30 min, cutting the test’s delivery time by two to three days as described in another study [34].

Gram-positive pathogens have consistently been difficult for MALDI-TOF to analyze. Some of them are challenging to distinguish because of their complicated composition of cell walls and the tight kinship between the species within the major genera. The success rate of identifying these pathogens at the species level has significantly risen as a result of the application of formic acid for a quick, on-plate protein extraction [54]. Despite significant advances in the databases that are provided, it is occasionally simple to distinguish between various *Streptococcus* species. An effort has been made to identify particular peaks that enable reliable distinction [55]. In addition, a method that uses the top 10 identifications obtained by MALDI-TOF has been created with the same objective [56]. With regard to anaerobes, MALDI-TOF has demonstrated to offer species-level identification compared with genetic methods in a quick and economical manner [57]. According to a multicentric study called ENRIA, which enabled the enhancement of the MBT database with approximately 6903 bacterial species and more than 60 distinct genera, significant advancements have been made in the accurate identification of anaerobes [58]. The upgraded database had a particularly big influence on the bacterial detection of Gram-positive anaerobic isolates, of which recognition was considered to be difficult due to the absence of spectral data from this category of microorganisms in earlier iterations of commercialized databases [19].

Mycobacteria are commonly recognized by MALDI TOF MS technology [24,25]. Unfortunately, this technology has the same drawback as real-time PCR in that it cannot distinguish between various Mycobacterium tuberculosis complex (MBTC) species [59]. Therefore, it is now unable to specifically identify tuberculosis species with this technology [60]. Since the advent of up-to-date, thorough datasets and the development of streamlined sample processing techniques in recent years, MALDI-TOF MS has been able to differentiate between a growing number of non-tuberculous Mycobacterium species. While MALDI-TOF MS is still able to differentiate between MBTC species in the coming year, this is still one of its greatest challenges [25]. It was demonstrated that the in vitro diagnostic method created by the company of Bruker Daltonics (Bremen, Germany), that uses crystal particles to mechanically interrupt the microbial biomass accompanied by ribosomal extraction, can provide identification at the species level [19]. Species with complex morphologies, for instance the *Mycobacterium avium*, can be successfully differentiated via a species-specific marker [61]. Overall, MALDI-TOF enables extremely accurate detection of *Mycobacteria* that are not tuberculous. The precision of this authentication is comparable to that offered by molecular biology techniques, although MALDI-TOF requires a response time of about one hour since the specimens must be heat-inactivated for half an hour [39]. Similar to mycobacteria, *Nocardia* and *Streptomyces* belonging to the order Actinomycetales must have their cell walls damaged in order for MALDI-TOF to correctly identify them. Because of their complex nomenclature, it can be difficult to differentiate between similar species.

### 4.2. Yeasts and Filamentous Fungi Identification

There are two major MALDI-TOF MS systems on the market today: the MBT (Bruker Daltonics) and the Vitek MS (BioMerieux). Although a distinct fungi database only became accessible for mold detection for experimental use in 2012, the FDA only approved the MBT in 2013 for the identification of bacteria and yeast. For bacteria and yeast identification, the FDA only approved the MBT in 2013 after a separate fungi database had been made available for experimental use in 2012. The FDA, however, approved the Vitek MS version 3.0 in 2017 with a library that included more bacteria, yeasts, mold, and mycobacteria [62]. In 2017, the FDA approved a version of Vitek MS containing additional bacteria, yeasts, molds, and mycobacteria. In spite of the fact that chances of success might vary greatly depending on microorganism type, database version, extraction process conditions, and threshold level, scientists have observed that both MBT and VITEK MS systems are capable of fairly excellent recognition of yeast [62]. In clinical microbiology labs nowadays, MALDI-TOF is applied routinely for the discovery of yeast species [18]. The identification of yeasts, particularly those from regularly occurring genera (e.g., *Cryptococcus* and *Candida*), is performed immediately using cultivated colonies on growth media and from medical samples, despite the fact that the utilize of protein extraction was recorded in earlier investigations. To identify non-*Candida* genera, most researchers recommend using internal databases.

Similarly, fungal species have been verified in the same way that nontuberculous mycobacteria have been verified: verification of these microbes usually requires prior disturbance to penetrate their cellular structure, the complexity of fungi is higher than that of bacteria, and the structure of protein molecules has changed over time as well. Until relatively recently, there were no comprehensive commercial databases to address these challenges. It is therefore not surprising to see a number of authors building their own internal libraries as a result of certain sample preparations [27]. There is currently no accepted standard procedure for detecting fungal species, but researchers working on MALDI-TOF have used equivalent mixtures of mechanical and chemical methods to solve this problem [63]. Eventually, Normand et al. [64] conducted a more extensive effort. To compare spectra from unidentified fungal isolates, a library with approximately 11,000 stored spectra from about 938 filamentous fungi of clinical significance has been created and made available on the web. *Aspergillus*, *Trichophyton*, and *Microsporum* species that are clinically meaningful can be correctly identified using the library, which enables microbiology labs to quickly and accurately carry out the necessary fungal identification [65,66].

### 4.3. Viral Identification

Numerous studies have investigated the use of MALDI-TOF MS to detect a wide variety of viruses, including Papillomaviridae, Picornavirus, and Orthomyxoviridae families [67]. As reported by Sjöholm et al. [68], MALDI-TOF MS had a reliability rate of 95.6% and a specificity of 98.0% when analyzing 882 fluid samples. With the aid of MALDI-TOF MS, Cai and co-workers developed a high-throughput genetic analysis method with a matching rate of 80.1% (285/356). Based on genetic analysis and MALDI-TOF MS, Peng et al. identified enteroviruses and 93.4% (225/241) of the findings were in agreement [69]. A MALDI-TOF MS analysis was also conducted to distinguish different influenza virus types, and the findings were 100% (29/29) compatible with genetic analyses [70]. To detect other respiratory viruses, such as influenza, in cell cultures of clinical samples, MALDI-TOF MS is used to analyze the protein spectral composition of the coat proteins of intact tobacco mosaic viruses [71,72,73]. The SARS-CoV-1 proteins and trypsin-digested peptides were additionally identified by MALDI-TOF MS [74]. This tool developed by Yoshinari and his coworkers combines simple purification processes with a rapid recognition phase to identify SARS-CoV-2-specific peptides accurately in nasopharyngeal swabs, which may offer a more accurate method of diagnosing COVID-19 in the future [71]. For COVID-19 testing, MALDI-TOF MS has been suggested as a viable approach [73,75]. Lazari and coworkers used MALDI-TOF protein signatures from salivary swabs to develop a new strategy for diagnosing COVID-19 and demonstrated that this technique could identify and predict the virus [76].

In the near future, clinical virology will benefit from the detection of resistant protein markers, the determinants of virulence, and MALDI-TOF MS applications for more direct detection of clinical samples. A change in working procedures would, however, be necessary if this technology were routinely used for virology diagnosis [77]. The fact that sensitivity can be identified, among other technical advancements, requires an increased level of integration into laboratory workflows. Diagnostics can be made immediately from clinical samples, and microorganism databases need to be updated continuously. MALDI-TOF should become available for everyday viral diagnostic testing as technological advancement plays a key role in viral identification through MS techniques.

### 4.4. Detection of Antibiotic Resistance

An urgent need exists for creative solutions to the rising rates of multidrug-resistant bacteria, not simply for treatment and preventive measures but also for the identification and characterization of isolates that are resistant to antibiotics [78,79]. The main barriers to starting a prompt targeted antibiotic therapy and/or infection prevention and control measures are the difficulty of conventional morphological sensitivity testing protocols and the limitations of quick DNA-based molecular diagnostics. An improved therapeutic results depend on receiving early, adequate treatment [80]. The benchmark standards for phenotypic antibiotic susceptibility testing (AST) depends on the identification of bacterial activity or suppression in the presence of an antibiotic. The currently offered conventional techniques of assessing AST take roughly a day to generate a result, despite the fact that AST seems to be most important since it can detect all types of resistance regardless of the mechanisms underlying that resistance [81].

New fast phenotypic AST methods are therefore required. Here, we introduce a freshly created technique known as a straightforward microdroplet growth test for the quick discovery of antibiotic resistance based on MALDI technology. In spite of the microbiological species, antimicrobial drug in issue, or fundamental resistance strategy, this method enables comprehensive morphologic AST, which enables quick separation between resistant and sensitive isolates [79]. Due to MALDI-TOF MS’s adaptability, increasing numbers of applications relying on this approach are beginning to appear. The identification of virulent biomarkers and the screening of antibiotic resistance are among the most intriguing ones currently being used or soon to be used in clinical settings [19]. Recently, MALDI-TOF MS is considered one of the most common methods used detecting antimicrobial resistance through measuring the hydrolysis of different antibiotics such as beta-lactamases and carbapenemases [82]. The beta-lactamase activities are conducted in a relatively similar manner across all the research papers [19]. An antibiotic buffer is reconstituted in fresh bacterial culture and incubated at 37 °C with stirring for one to two hours. As soon as the culture period is over, the mixture is spun, and the supernatant is transferred to a MALDI-TOF work-piece to be analyzed. Samples can be inserted, dehydrated, and then analyzed by MALDI-TOF MS. Researchers have reported that cephalosporins have the spectra of cefotaxime, ceftazidime, ceftriaxone, cefpodoxime, and cefepime as well as their hydrolysis products [53]. Because of cefepime’s limited sensitivity, which causes false positive results in the hydrolysis, it has been rejected for MALDI-TOF identification of beta-lactamases, while ceftriaxone is the most reactive, selective, and fastest [19].

**Figure 2:**
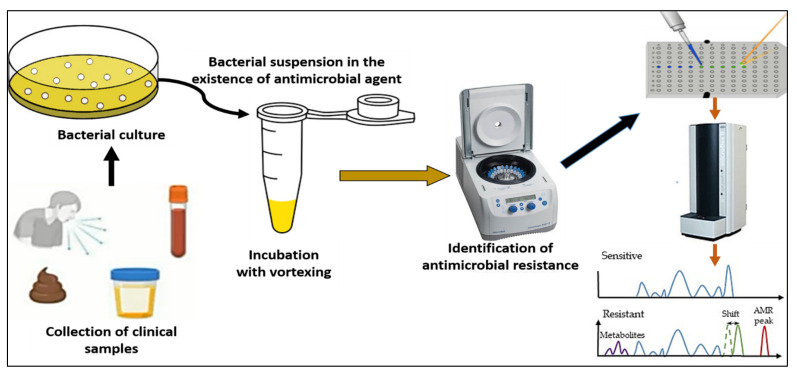
A workflow for MALDI-TOF detection of antimicrobial resistance.

Extended spectrum beta-lactactamases (ESBL) producing bacteria would exhibit positive degradation after 30 min of incubation (Figure 2). Although cefotaxime has more expertise, the outcomes for cefotaxime and cefpodoxime are comparable. Both have susceptibility levels exceeding 90%, however for both, a 1 h incubation period is advised. Ceftazidime is not advised as a drug of choice for the investigation of beta-lactactamases since it significantly reduces the efficiency of hydrolysis and, subsequently, MALDI-TOF MS recognition. The suggested incubation period of three hours is based on the susceptibility being less than 90% [19].

With the aid of MS, it has been described how ertapenem, meropenem and imipenem are hydrolyzed in the context of carbapenem antibiotics. A 2 h incubation period is recommended for both ertapenem and meropenem due to their high sensitivity. Mass spectra of ertapenem show clearly defined peaks, however mass spectra of meropenem and imipenem are excluded [83]. It has been suggested that dihydroxybenzoic acid could be used as a matrix for analyzing meropenem and its hydrolyzed product [84]. The MBT STAR-BL IVD software, used in conjunction with the MBT STAR-Carba IVD Kit and the MBT STAR-Cepha IVD Kit, is a tolerance package for the MBT. The program automatically analyzes peaks and produces a merged report including the verification of the strain of bacteria and its imipenem sensitivity. Using this technology, even non-MS specialists can evaluate hydrolysis as it is automated [19]. In order to differentiate sensitive from resistant bacteria of the same species, the peak evaluation method is used. The peaks can be allied with a particular design if the transcription of a protein or peptide is related to the resistance phenotype directly or indirectly. By comparing mass peaks, the user can determine which germs are vulnerable and which ones are resistant. Using the spectra of beta-lactamase as a marker, Camara and Hays [85] distinguished between ampicillin-resistant strains of plasmid-transformed *E. coli* from different sources. MALDI-TOF MS was successfully used in 2011 to distinguish between *Bacteroides fragilis* that are *cfiA*-positive and *cfiA*-negative, and subsequently their carbapenem resistance. In spite of this, these examinations still seem to suggest additional incubation time, albeit less than for antibiograms, which intrinsically delays assessment of which antibiotherapy will work best. Before MALDI-TOF MS can be regarded as the new reference approach for the screening of antibiotic resistance in regular investigations, there remains a great deal of work concerning our review on the identification of antimicrobial resistance by specialized MALDI-TOF spectra patterns. There are still a lot of unanswered problems, thus further research on pathogenic organisms is needed. The scientists need to be very interested in research on critically important antibiotics like cephalosporins or macrolides, which are frequently utilized to treat infections in hospital settings despite the lack of evidence on them. Last but not least, the combined strategy of molecular and MALDI-TOF MS should quickly replace other methods as the primary method for the most accurate and quick diagnosis of antimicrobial resistance in clinical microbiology labs.

### 4.5. The Advantages and Drawbacks of MALDI-TOF MS Technology

It is challenging to distinguish between organisms that exhibit similar phenotype, biochemical, and even genetic characteristics when using conventional or molecular techniques. A wrong species identification may result from grouping closely related species (such as coagulase-negative staphylococci). MALDI-TOF MS, however, analyzes microbial proteins, which are often highly conserved within a species, so it can differentiate between species with greater accuracy. Numerous investigations have demonstrated that MALDI-TOF MS can reliably and frequently discriminate between microbial pathogens that are strongly related [3,86,87]. For microorganisms where a misidentification or a dearth of a species-level identification may have a clinically meaningful effect, this is especially beneficial. This could apply to species that have few therapeutic strategies, are consistently resistant to a particular group of antibiotics, or in which physicians make treatment recommendations based only on diagnosis because sensitivity testing is not regularly carried out [3]. Among the bacteria with extremely complex nutrient requirements, including staphylococci with coagulase-negative, streptococci with nutrient variants, and pathogens from the HACEK family (*Haemophilus*, *Aggregatibacter*, *Cardiobacterium*, *Eikenella*, *Kingella*), conventional methods are hard to identify at the species level but MALDI-TOF MS can be used to detect them easily. As an example, one investigation found that MALDI-TOF MS consistently detected over 86% of HACEK isolates, whereas biochemical testing recognized only 77% [86].

Identifying pathogens in a timely manner is vital when an infection is unforeseen, which is possible with MALDI-TOF MS. The incident report by von Rotz et al. [88] describes a father and son who were hospitalized with sepsis and gastroenteritis following camping. MALDI-TOF MS was able to identify *Listeria monocytogenes* (*L. monocytogenes*) right from the blood culture container, within an hour of taking blood cultures and testing them for Gram-positive rods. As a foodborne pathogen, *L. monocytogenes* is seldom encountered in humans, but it tends to infect pregnant women, those with compromised immune systems, and the elderly. In the case of patients who have been identified, the appropriate medicines have been prescribed, and the patients have recovered without complication. The detection of anaerobes and yeast is another example of how MALDI-TOF MS can improve turn-around times. A large amount of biomass is required for accurate biochemical identification of anaerobic species because of their slow doubling times and lack of biochemical activity. Conventional techniques require a substantial amount of time to carry out analytical tests rather than growing the organism for final identification, thus causing substantial delays in diagnosis. Faster detection of yeasts can greatly enhance treatment benefit. There are several types of aggressive yeast infections, including *Candida albicans*, *Candida glabrata*, *Candida krusei*, and *Candida parapsilosis*, that differ in sensitivity patterns and pose a high mortality and morbidity risk to immunocompromised individuals [89]. The onset of empiric therapy can be shortened as a result of prompt identification because antifungal susceptibilities are predictable for different species. Clinical outcomes have been demonstrated to be improved, and hospital stays to be shortened as a result [90,91].

Diagnostic laboratories can save a considerable amount of money by implementing MALDI-TOF MS as the primary identification technique. This device, although expensive, can quickly pay for itself through the savings on reagents and labor. A lab that runs a moderate to high volume of tests would likely save more than 50% in consumables [15,92]. In light of the fact that some species may take longer to identify using MALDI-TOF MS [15], labor reductions are likely to be limited. Technological advancements and the ability to accurately identify more species have the potential to result in even more cost savings.

Although most closely related species of organism frequently found in clinical laboratories can be reliably identified by MALDI-TOF MS, there are still some limitations of using this technology. Due to the similarity of microorganisms themselves, it may not be able to distinguish between closely related species. Several biologists have suggested that *Echerichia coli* and *Shigella* may potentially be the same species, explaining why MALDI-TOF MS is not able to distinguish between them [93]. MALDI-TOF MS has nevertheless been claimed to be capable of detecting differences between these species [94,95]. In addition, *Burkholderia cereus*, *Burkholderia cepacia*, *Burkholderia mallei/pseudomallei*, *Achromobacter* species, *Citrobacter freundii*, *Enterobacter cloacae*, *Salmonella* [96], *Mycobacterium tuberculosis*, *Mycobacterium abscessus*, and *Mycobacterium avium* are among the other examples [97,98]. Generally, microorganisms that are intrinsically related are reported as a group, complex, or genus. In cases where species-level differentiation is clinically important, additional testing should be conducted. MALDI-TOF MS may be enhanced in the future with proteomic-based techniques to increase discriminatory strength and allow identification of organisms down to the strain or serotype level [97].

Another factor that could make identical species appear to be misidentified is the lack of sufficient spectra in the database. There is the potential to receive an accurate detection that is not accurate or to not receive any recognition at all in these circumstances. It has been found that similar *Trichophyton* species are usually misidentified [99]. It is also possible to misidentify species when not all members of a species complex are included in the database. An investigation by Body et al. found that most isolates of *Mycobacterium mucogenicum* were correctly identified, but *mycobacterium phocaicum*, which was not in the database, was frequently confused with *Mycobacterium mucogenicum* [98]. These errors are not always a cause for concern, but can sometimes have a significant impact on patient care. Due to their different degrees of macrolide resistance, *Mycobacterium abscessus* subspecies such as abscessus and bolletii and massiliense cannot be distinguished. Some anaerobes, like *Bacteroides*, *Fusobacterium*, and *Lactobacillus* species, have been able to overcome this problem through database updates or user-created libraries. It can be successful to use backup techniques like sequencing if the problem is known.

## 5. Outlooks for the Future

Clinical uses of MS are becoming an increasingly popular area of research with enormous implications for public health. One of the most complex improvements has been made by carefully coordinating molecular techniques with MALDI-TOF. The best way to detect complex genetic markers without involving sequences is through MS, as it is a revelation approach [42]. By developing MS-based molecular techniques, new methods will be developed for retrieving important genetic information experimentally and from outbreaks by utilizing primarily advanced mathematical and computational models, including the intensity of disorders, medication resistance, vaccination retreat, and transmission [32]. It will be critical to the success of the MS invention to be able to rapidly identify microorganisms responsible for hospital infections [24]. As long as appropriate rapid and delicate current technology is used for the medical detection of infectious diseases, MS can significantly affect the quality management of sterile blood items and food security. As part of the integration of molecular and computational approaches with MS, analytical assays have to be developed for numerous, ongoing applications in public health and clinical practice [100].

In conclusion, MALDI-TOF mass spectrometry technology is one of the most widely used reference methods for identifying various types of microorganisms including viruses and antimicrobial resistance. Using this technology for identifying antibiotic resistance has also gained widespread acceptance in healthcare settings, improving turnaround times and streamlining workflow. Currently, MALDI-TOF data needs to be confirmed with slower molecular techniques in order to detect virulence markers or bacterial typing, but it is likely MALDI-TOF will soon replace first-line technologies. When used in conjunction with other cutting-edge technologies, such as molecular techniques, MALDI-TOF can quickly and affordably send messages that have a therapeutically significant impact. Due to its speed, accuracy, sensitivity, and high throughput, MALDI-TOF MS is anticipated to play a more significant role in the advancement of clinical microbiology in the future.

## Data Availability

Not applicable.
